# Pancreatico-Jejunostomy On Isolated Loop After Pancreatico-Duodenectomy: Is It Worthwhile?

**DOI:** 10.1007/s11605-022-05296-y

**Published:** 2022-03-16

**Authors:** Gennaro Clemente, Agostino Maria De Rose, Elena Panettieri, Francesco Ardito, Marino Murazio, Gennaro Nuzzo, Felice Giuliante

**Affiliations:** Hepatobiliary Surgery Unit, Fondazione Policlinico Universitario Agostino Gemelli IRCCS, Università Cattolica del Sacro Cuore, Rome, Italy

**Keywords:** Pancreatico-duodenectomy, Pancreatico-jejunostomy, Isolated-loop pancreatico-jejunostomy

## Abstract

**Background:**

Postoperative morbidity remains a significant problem after pancreatico-duodenectomy. The management of pancreatic stump continues to be a challenge, and many technical solutions have been developed over the years. In this study, we report the results obtained with the use of an isolated loop for pancreatico-jejunostomy in patients with soft pancreas and small pancreatic duct diameter.

**Methods:**

Clinical data of patients submitted to pancreatico-duodenectomy in a period of sixteen years (2005–2020) were extracted from a prospective database. Patients with soft pancreas, main duct diameter < 2 mm and reconstruction by pancreatico-jejunostomy on single loop or isolated loop were selected. Primary end-point was the incidence of clinically relevant fistulas in the two groups of patients. Secondary endpoint was the length of postoperative hospital stay. A propensity score matching analysis was used for the statistics.

**Results:**

Two hundred and twenty-one patients with the above characteristics were found in the database. One hundred and twelve of these received a single-loop reconstruction and 109 an isolated loop reconstruction. Incidence of clinically relevant fistulas was higher in the first group (41% vs 27%; *p* = 0.023). Postoperative hospital stay was significantly shorter in the second group (21 days vs 15; *p* < 0.001). These results were confirmed at the propensity score matching.

**Conclusion:**

In patients with soft pancreatic texture and small main duct diameter, pancreatico-jejunostomy on isolated loop is associated with a lower incidence of clinically relevant fistulas than after classic reconstruction. The duration of postoperative hospital stay was significantly reduced, with consequent reduction of cost.

## Introduction

Pancreatico-duodenectomy (PD) represents today the standard treatment of the periampullary neoplasms (cancers of the head of the pancreas, ampulla, distal common bile duct and periampullary duodenum). In recent years, the advances in surgical technique and postoperative care have led to a significant decrease in mortality of this procedure to less than 2%. ^1^ Despite notable improvements in mortality, morbidity remains high, not less than 35%, also in several series from high-volume centers. ^2^ Complications are mainly related to the pancreatico-jejunal anastomosis (PJ) with postoperative pancreatic fistula (POPF) being the most dreaded complication and the major potential cause of mortality. ^3,4^ Consequently, the management of pancreatic stump continues to be a challenge for pancreatic surgeons, and many technical solutions have been developed over the years in order to reduce the incidence of POPF. However, the absence of an unanimous agreement on the best technique indicates that the issue is far from being resolved. One of the proposed techniques is the accomplishment of the PJ on an isolated intestinal loop; this reconstructive technique was introduced by Machado in 1976. ^5^ The rationale of the Machado’s procedure is to separate the pancreatic juice from bile and gastrointestinal secretions, in order to maintain inactive the pancreatic juice in case of POPF and to avoid the catastrophic consequences that, starting from the infection of the surgical site, compromise the patient's life. Results of this technique reported in the literature are conflicting to date. However, it is ascertained from the literature that the occurrence of POPF is strictly related to soft pancreatic texture and to small pancreatic duct diameter. ^6^ Consequently, the use of an isolated PJ loop could be useful at least in this group of patients. Validation of this hypothesis represented the main purpose of this study. In this paper, we reported the incidence and severity of POPF after isolated-loop reconstruction (ILR) versus conventional reconstruction (CR) in patients with soft pancreatic texture and small pancreatic duct diameter. Primary end-point of this study was the incidence of clinically relevant POPF in that population. Secondary endpoint was the length of postoperative hospital stay.

## Patients and Methods

### Selection of patients

All consecutive patients with pancreatic-biliary malignancies candidate for radical PD were entered in a prospectively maintained database, including both clinical- and surgery-related data. The former were: age and sex, BMI, site of the tumor, preoperative biliary drainage, radiological staging examinations performed. The latter were: type and duration of surgical procedure, blood loss, pancreas texture (hard or soft), diameter of the main pancreatic duct, type of PJ reconstruction, postoperative medical therapy, postoperative complications including POPF, delayed gastric emptying (DGE), postpancreatectomy hemorrhage (PPH), need for postoperative interventional radiology procedures, re-laparotomy, length of hospital stay, mortality.

Inclusion criteria were:

- standard PD, with or without pylorus preserving (Whipple or Traverso procedure);
- soft pancreatic texture with main duct diameter < 2 mm;
- reconstruction by PJ with “duct-to-mucosa” technique or “stump invagination” technique;
- standard postoperative therapy with a somatostatin analogue (octreotide) administration for at least seven days (0.2 mg/ml three times a day).

Among these patients, two groups were then identified: those with PJ on the same loop of the hepatico-jejunostomy and gastro-jejunostomy (CR group) according to Child ^6^ and those with PJ on an isolated-loop (ILR group) according to Machado. ^5^ In each group, the sex, the age of the patients, the BMI, the site of the neoplasm, any preoperative biliary drainage, the diameter of the main pancreatic duct, the type of demolition (Whipple or Traverso) and the type of anastomosis (duct-to-mucosa or stump invagination) were noted. Duration of surgery, blood loss, morbidity and mortality at 90 days were then compared in the two groups. Complications were classified according to the ISGPS criteria. ^7–9^ In particular, for definition and grading of POPF, the 2016 updated criteria of the ISGPF ^10^ were used: biochemical leak, grade B and grade C (the two latter being clinical relevant fistulas). The following data were also compared: the need for interventional radiology, re-laparotomy, length of postoperative hospital stay and mortality at 90 days.

### Statistical Analysis

For comparison of categorical and quantitative variables, Fisher’s exact test and two-tailed unpaired t-test (normally distributed data) or a Mann–Whitney U test (not normally distributed data) were used, respectively. *p* < 0.05 was considered statistically significant. Categorical data were expressed as patient number and percentage of the respective patient cohort. To overcome inherent imbalances between the groups, we performed a propensity score matched analysis between CR group and ILR group using the nearest-neighbor method to 1:1 ratio. Propensity score deviation width was set to a threshold of < 0.2. Variables used for matching were classical relevant factors predicting postoperative clinically relevant POPF: pancreatic cancer vs other tumors, BMI, main duct diameter (1 vs 2 mm) and intraoperative blood (< 400 ml; 400–700 ml, 700–1000 ml). To detect residual imbalances after matching, bivariable and multivariable binary logistic regression analyses were conducted to identify factors associated with clinical relevant POPF. Variables that showed statistically significant association (*p* < 0.05) in the bivariable binary logistic regression are entered in the multivariable model. Adjusted odds ratio (aOR) along with 95% CI was estimated to identify factors independently associated with clinical relevant POPF. All analyses were carried out with SPSS software version 25 and STATA software version 14.

## Results

**Fig. 1:**
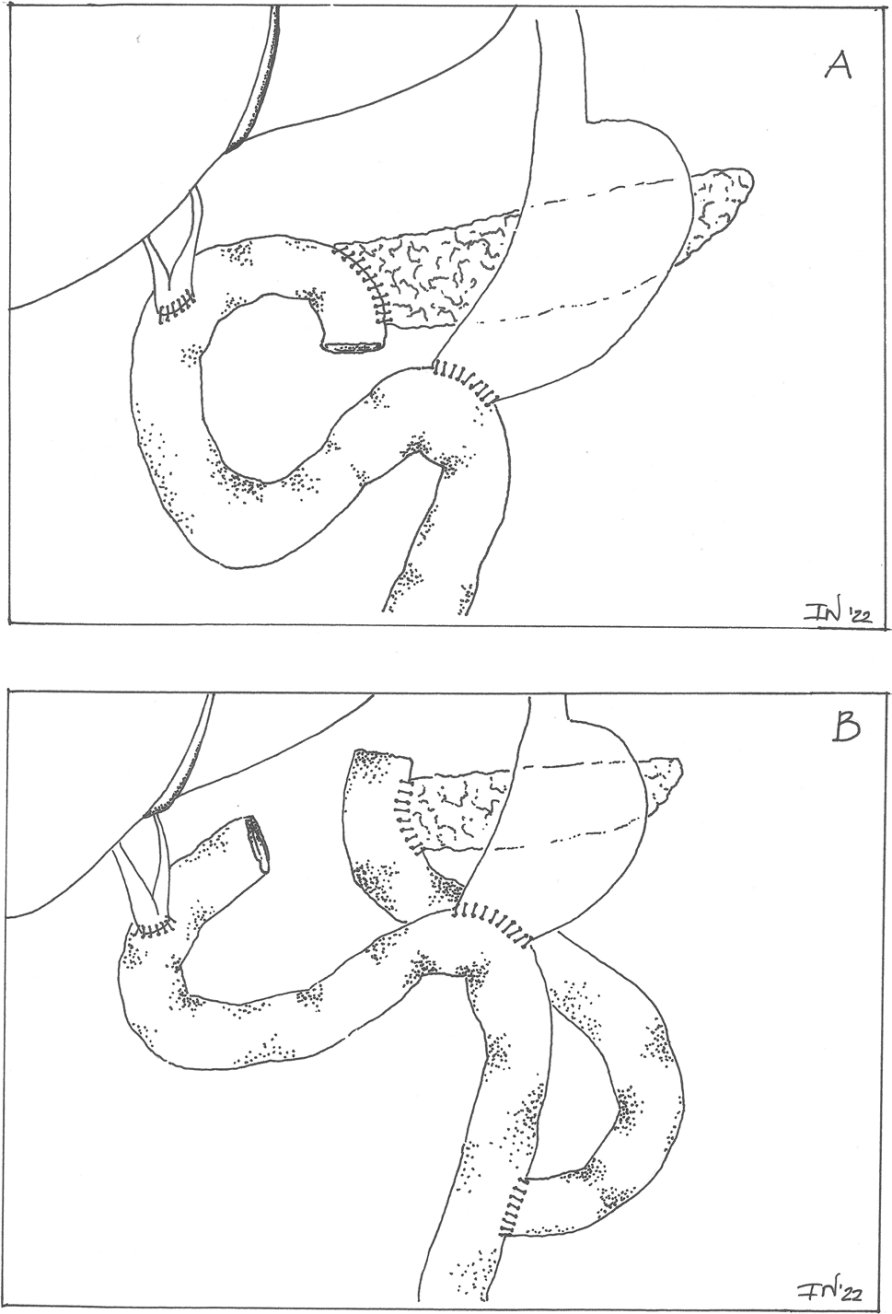
Single-loop reconstruction (**A**) and double-loop reconstruction (**B**)

Between January 1, 2005, and December 31, 2020, 384 patients underwent pancreatic resection for neoplasm at our unit. Two hundred and twenty-one of these fulfilled the inclusion criteria for this study. One hundred and twelve patients received a single-loop reconstruction (CR group) according to the Child technique ^6^ and 109 received a double-loop reconstruction (ILR group) according to Machado.^5^ In patients of the CR group, PJ was performed firstly, transposing the first jejunal loop through the mesocolon, followed approximately 30 cm downstream by the hepatico-jejunal anastomosis and then by the gastro-jejunostomy, or duodeno-jejunostomy. In patients of the ILR group, the first jejunal loop was isolated for a length of 60 cm: the head of the loop was transposed through a separate opening into the mesocolon and anastomized to the pancreas; the distal head was anastomosed to the efferent loop 60 cm downstream of the gastro-jejunostomy, or duodeno-jejunostomy. The surgical procedures performed in the two groups of patients (CR and ILR) are illustrated in Fig. 1. In all cases, a double layer of interrupted stitches was made, using a long-term adsorbable material. An internal stent between the pancreatic duct and the jejunum was left in place in all patients of both CR group and ILR group. In addition, three drains were placed in all patients: the first near the biliary anastomosis and other two near the pancreatic-jejunostomy. The amylase value in the drainage fluid was routinely determined on the first and third day; if the amylase value was normal (less than three times the serum amylase), the drains were removed starting from the fourth day. Otherwise, the amylase value was repeated every other day and the last drain was removed when the value was normal. In case of POPF without serious consequences, the treatment was initially conservative. If CT revealed peripancreatic collections, CT-guided drainage was performed. In case of deterioration of the clinical status with sepsis and organ dysfunction, the patient was re-explorated: In the majority of cases, a demolition of the PJ with preservation of the pancreas remnant and external drainage of the pancreatic juice were performed. All surgeries were performed by four senior surgeons (GC, MM, GN and FG) with expertise in pancreatic surgery. Octreotide was administered as prophylaxis of the POPF for at least seven days in all patients.

Demographic, clinical, surgical and postoperative characteristics are reported in Table 1. The overall incidence of POPF and the incidence of clinically relevant POPFs were significantly higher in the CR group (*p* = 0,017 and *p* = 0.023, respectively). The need for interventional radiology was also significantly higher in the CR group (*p* = 0.012). The postoperative length of hospital stay was significantly shorter in patients of the ILR group (15 ± 5 vs 21 ± 12 days; *p* < 0.001). Results after propensity score matching are reported in Table 2 (96 patients for each group): clinically relevant POPF (*p* = 0.015), need for interventional radiology (*p* = 0.007) and postoperative hospital stay (*p* = 0.001) were significantly lower in the ILR group. Table 3 shows the results of the uni- and multivariable logistic regression analysis for factors predicting clinically relevant POPF: male sex, small diameter of the main duct, intraoperative blood loss and conventional reconstruction (CR) were statistically significant predictors of POPF development in our series.

**Table 1:** Clinical and Surgical Data of 221 Patients, with “Soft” Pancreas and Small Diameter of The Main Duct, Who Underwent Pancreatico-duodenectomy with Conventional Reconstruction (112 pts.) or Isolated-Loop Reconstruction (109 pts.)

Data	Conventional Reconstruction (*n* = 112)	Isolated-Loop Reconstruction (*n* = 109)	*p*
Patient factors			
Age, mean (SD), yr	62.02 (11.16)	63.44 (10.85)	0.243
Sex, n (%)			
Male	64 (57.1)	58 (53.2)	0.557
Female	48 (42.9)	51 (46.8)	
BMI, mean (SD)	24.95 (± 2.76)	26.43 (± 2.06)	< 0.001
BMI ≥ 25 n (%)	56 (50)	69 (63)	0.046
Site of tumor			
Pancreas, n (%)	39 (34.8)	38 (34.9)	0.995
Other tumors, n (%)	73 (65.2)	71 (65.1)	
Common bile duct, n (%)	34 (30.4)	32 (29.4)	0.871
Ampulla, n (%)	32 (28.6)	34 (31.2)	0.670
Duodenum, n (%)	7 (6.2)	5 (4.6)	0.585
Preoperative biliary drainage	77 (68.7)	75 (68.8)	0.877
Wirsung diameter, mean (SD), mm	1.33 (0.47)	1.45 (0.49)	0.070
Surgical factors			
Whipple procedure, n (%)	57(50.9)	58 (53.2)	
Pylorus preserving, n (%)	55(49.1)	51(46.8)	0.730
Type of anastomosis			
Duct-to-mucosa, n (%)	92(82.1)	82 (27)	0.209
Stump invagination, n(%)	20 (17.9)	27 (24.8)	
Duration of surgery, mean (SD), minutes	507.06 (61.82)	527.89 (56.65)	0.010
Intraoperative blood loss, n (%)			
< 400 ml	96 (85.7)	92 (84.4)	0.783
400 – 700 ml	13 (11.6)	15 (13.8)	0.630
700 – 1000 ml	3 (2.7)	2 (1.8)	0.673
Postoperative outcomes			
Postoperative stay, mean (SD), days	21.31 (11.65)	14.96 (4.66)	< 0.001
90-day mortality, n (%)	3 (2.7)	3 (2.7)	0.973
Postoperative complications, n (%)			
POPF	79 (70.5)	60 (55)	0.017
Biochemical leak	33 (29.5)	31 (28.4)	0.867
Clinically relevant POPF	46 (41.1)	29 (26.6)	0.023
Delayed gastric emptying	53 (47.3)	43 (39.4)	0.238
Postoperative pancreatic hemorrhage	5 (4.5)	8 (7.3)	0.364
Re-laparotomy, n (%)	16 (14.3)	14 (12.8)	0.836
Interventional radiology, n (%)	42 (37.5)	24 (22.0)	0.012

Abbreviations: SD, standard deviation, POPF, postoperative pancreatic fistula

**Table 2:** Clinical and Surgical Data of 192 Patients, with “Soft” Pancreas and Small Diameter of the Main Duct, Who Underwent Pancreatico-duodenectomy with Conventional Reconstruction (96 pts) or Isolated- Loop Reconstruction (96 pts) after Propensity Score Matching

Data	Conventional Reconstruction (*n* = 96)	Isolated-Loop Reconstruction (*n* = 96)	*p*
Patient factors			
Age, mean (SD), yr	62.07 (11.08)	63.44 (10.78)	0.620
Sex, n (%)			
Male	56 (58.3)	50 (52.1)	0.384
Female	40 (41.7)	46 (47.9)	
BMI, mean (SD)	25.59 (± 2.09)	25.82 (± 2.39)	0.254
BMI ≥ 25 n (%)	56 (58)	57 (59)	0.883
Site of tumor			
Pancreas n (%)	37 (38.5)	38 (39.6)	0.882
Other tumors, n(%)	59 (61.5)	58 (60.4)	
Common bile duct, n (%)	24 (25.0)	25 (26.0)	0.868
Ampulla, n (%)	29 (30.2)	30 (31.3)	0.876
Duodenum, n (%)	6 (6.3)	3 (3.1)	0.306
Preoperative biliary drainage	65 (67.7)	66 (68.8)	0.877
Wirsung diameter, mean (SD), mm	1.39 (0.49)	1.39 (0.49)	1.000
Surgical factors			
Whipple procedure, n (%)	51(53.1)	50 (52)	0.885
Pylorus preserving	45(46.9)	46(48)	
Type of anastomosis			
Duct-to mucosa	78 (81.2)	72 (75)	0.295
Stump invagination	18 (18.8)	24 (25.0)	
Duration of surgery, mean (SD), minutes	505.41 (62.03)	529.54 (58.43)	0.657
Intraoperative blood loss, n (%)			
< 400 ml	82 (85.4)	82 (85.4)	
400 – 700 ml	13 (13.5)	13 (13.5)	1.000
700 – 1000 ml	1 (1.0)	1 (1.0)	
Postoperative outcomes			
Postoperative stay, mean (SD), days	21.54 (12.26)	15.14 (4.56)	0.001
90-day mortality, n (%)	2 (2.1)	2 (2.1)	1.000
Postoperative complications, n (%)			
POPF	66 (68.7)	54 (56.2)	0.074
Biochemical leak	25 (26.0)	29 (30.2)	0.521
Clinically relevant POPF	41 (42.7)	25 (26.0)	0.015
Delayed gastric emptying	44(45.8)	40 (41.7)	0.561
Postoperative pancreatic hemorrhage	4 (4.2)	7 (7.3)	0.352
Re-laparotomy	14 (14.6)	13 (13.5)	0.836
Interventional radiology, (n%)	37 (38.5)	20 (20.8)	0.007

Abbreviations: SD, standard deviation, POPF, postoperative pancreatic fistula

**Table 3:** Univariable and multivariable logistic regression analysis for factors predicting clinical relevant POPF

Parameter		Univariate Analysis		Multivariate Analysis	
		P	cOR (CI95%)	P	aOR (CI95%)
Age	≥ 65 vs. < 65 years	0.924	0.972 (0.535 – 1.763)		
Sex	Male vs Female	0.004	2.530 (1.346 – 4.754)	0.017	2.381 (1.168 – 4.854)
BMI	< 25 vs ≥ 25	0.028	2.011 (1.077 – 3.756)		
Tumor Site	Other tumors vs pancreatic cancer	0.704	0.889 (0.484 – 1.633)		
Wirsung Diameter	1 mm vs. 2 mm	< 0.001	6.749 (3.078 – 14.796)	< 0.001	6.406 (2.784 – 14.753)
Surgical Technique	Double-loop vs. Single-loop	0.016	0.472 (0.257 – 0.869)	0.011	0.399 (0.197 – 0.810)
Intraoperative blood loss	≥ 400 vs. < 400 ml	< 0.001	6.413 (2.639 – 15.583)	0.002	4.845 (1.820 – 12.896)

cOR: crude odds ratio; aOR: adjusted odds ratio; CI: confidence intervals

## Discussion

Reconstruction after PD using an isolated Roux loop for PJ was described for the first time by Marcel D.C. Machado in 1976. ^5^ The Author used this technique in 15 patients, with two cases of POPF that healed twenty days after surgery. The mortality was nil. Over ten years later, in 1987, Funovics ^11^ published in the same Journal the results of a study carried out comparing end-to-side PJ (33 patients, 15% mortality), end-to-end PJ (31 patients, 6.5% mortality) and PJ on an isolated loop (48 patients, 2% mortality). The Author defined the separation between the bile loop and the pancreatic loop, the best option to prevent dangerous complications of POPF. Despite the good results reported by Machado and Funovics, this reconstructive technique was not extensively used in the following years. In 1994, Kingsnorth ^12^ reported the results obtained in 52 patients using an isolated loop for PJ, without any POPF. In 2002, the same results were reported by Khan ^13^ in 41 cases and by Sutton ^14^ in 61 patients in 2004. These Authors also pointed out that the absence of POPFs is the consequence of the removal of bile and gastric secretions from PJ. Undoubtedly, these results appeared particularly interesting, and in the following years further experiences were reported, not always in agreement with those reported by the aforementioned Authors.

In 2008, Grobmyer et al. ^15^ reported a retrospective study, performed on 700 patients treated between 1991 and 2006, comparing three different types of reconstruction after PD: PJ on isolated Roux loop (type A); gastro-jejunostomy on isolated Roux loop with PJ and hepatico-jejunostomy on the same loop (type B); conventional reconstruction with all the anastomoses on the same loop (type C). In the Grobmyer’s study, there was no difference regarding postoperative morbidity and mortality between these three groups. The overall incidence of POPF was 7.2%, and in patients with POPF, the mortality rate was 6%. In the Grobmyer’s study, there was also no difference in the need for percutaneous drainage and in the percentage of reoperations. The postoperative hospital stay was similar among the three groups. The conclusions of Grobmyer ^15^ were that the use of an isolated Roux loop after PD does not benefit, and it is therefore unnecessary. Nevertheless, some remarks are needed: the A group (PJ on isolated loop) included only 12 patients, the group B included 100 patients and the group C included 588 patients. In addition, all patients of this study were treated for cancer of the pancreas. In practice, the PJ on isolated loop was used in very few patients and, moreover, in patients with pancreatic parenchyma supposedly "hard" and therefore at low risk of POPF.

In 2015, Klaiber and **co-workers** ^16^ reported a meta-analysis study performed using three RCTs ^17–19^ and four CCTs. ^20–23^ Conclusions were that dual-loop reconstruction is not superior to single-loop reconstruction: no significant statistical differences were found regarding pancreatic fistula and other complications. However, in one of the three RCTs ^19^ analyzed by Klaiber, the dual-loop reconstruction is compared with the pancreatico-gastrostomy, and therefore, if the purpose of the meta-analysis is to compare single-loop and double-loop reconstruction, this trial should not be considered. The two remaining RTCs effectively compared single-loop and dual-loop technique and are the studies of Tani et al. ^17^ and Ke et al. ^18^. In the first study, 153 patients were randomized to receive single-loop (76 patients) or dual-loop reconstruction (77 patients). POPF occurred in 26 patients of the first group and in 25 patients of the second group; a relevant POPF (grade B or C) occurred in 10 and 11 patients, respectively. Soft pancreas resulted the only independent risk factor for POPF. Ke ^18^ reported the results of a prospective randomized multicenter study. From January 2006 to April 2012, 216 patients were enrolled: 107 of these received a reconstruction with isolated loop and 109 a reconstruction with single loop. The overall incidence of POPF was similar between the two groups (16% versus 18%, respectively), but the incidence of clinically significant POPF was lower after dual-loop reconstruction, and these patients had a shorter hospital stay and lower hospital costs. The most important risk factor in determining the fistula was the tumor site: the duodenum or the ampulla, usually associated with a “soft” texture of the pancreas. Both studies are well conducted, but they reach opposite conclusions. How can we explain these different results between these studies? In the study of Ke ^18^, the majority of patients had a soft pancreas: 136 / 216 = 63%, while in the Tani’s series ^17^ the soft pancreas was found in 69 / 151 patients = 45.6%. Since advantages of the dual-loop reconstruction could be particularly evident in patients with a soft pancreas, these data may partly explain the different results. Moreover, in Tani’s series the average loop length was shorter (40 cm) if compared to Ke's series, in which a greater length (60 cm) of the isolated loop was used. The choice of a very short isolated loop is pointed out by Tani et al. ^17^ to explain the results of their study.

The four CCTs used by Klaiber ^16^ for his meta-analysis are the studies of Ballas et al. ^20^, Kaman et al. ^21^, Casadei et al. ^22^ and Perwaiz et al. ^23^. In the Ballas study ^20^ that includes 88 patients, 42 classical reconstruction were compared with 46 reconstructions on an isolated loop, but no advantages were found with the second technique, which instead lengthen the average time of surgery of at least 30 min. However, in this study, the criteria of POPF are not clearly defined and patients with POPF are not divided into the categories of the ISGPF. ^7^ The study of Kaman and co-workers ^21^ included 111 patients submitted to Whipple procedure, with PJ on a single loop in 51 cases and on a double loop in 60 cases. The Authors do not report any difference regarding morbidity and mortality between the two groups but emphasize the greater time required (on average one hour and 18 min) and the increased need for transfusion using the isolated-loop technique. The latter data are questionable, and overall mortality in this study was too high (8%). In the study of Casadei and co-workers ^22^ regarding 38 consecutive patients submitted to PD with reconstruction on single loop (20 pts.) or dual loop (18 pts.), there were no significant differences in postoperative outcome between the two groups, but the isolated Roux loop reconstruction allowed for a significant decrease in length of postoperative hospital stay. The study of Perwaiz ^23^ was a retrospective study on 108 patients, 53 of whom underwent reconstruction with PJ on isolated loop and 55 underwent the classical reconstruction on a single loop. The results were similar in the two groups regarding mortality (2 patients per group), morbidity and duration of hospitalization; the only difference regards the duration of surgery (442 min for the isolated loop and 370 min for classical reconstruction, with a statistically significant difference: *p* = 0.005). Conclusions of the study were that the use of an isolated loop for pancreatic anastomosis is useless and entails a significant increase in operative time (70 min on average, for the small intestine anastomosis). However, in the Perwaiz study ^23^, as in the Kaman study ^21^, POPFs are not classified according to the ISGPF criteria. ^7^ Recently, MCC Machado ^24^ reported 214 consecutive patients treated with dual-loop technique and emphasized the advantages of a systematic use of isolated pancreatic anastomosis for reconstruction after PD. Aghalarov et al. ^25^ proposed a modification of the single-loop technique to separate bile and pancreatic juice. The technique proposed by Aghalarov does not influence the rate of POPF, but reduced its severity, leading to less major morbidity and mortality. More recently, Lyu et al. ^26^ reported a systematic review and meta-analysis comparing isolated PJ, isolated gastro-jejunostomy, and conventional PJ after PD. The results of this meta-analysis showed that isolated PJ was associated with a lower reoperation rate, but required longer operation time vs conventional PJ.

After analysis of the available literature, it is clear how problem of the isolated loop reconstruction after PD is still far from being solved. It is likely that the different results reported in the literature reflect different categories of patients examined. Since the incidence of POPF is higher in pancreas with soft texture, we tried to verify whether there may be an indication for ILR in this particular clinical condition. In our study, having the limitations of a retrospective study, although based on a prospective database, we tried to verify the usefulness of double-loop reconstruction in patients with soft pancreatic texture and small-diameter main pancreatic duct. In fact, in the majority of our cases, the sites of tumors were the ampulla, the distal CBD or duodenum, which are more frequently associated with the soft texture of the pancreas. Analysis of results showed that, in patients with the aforementioned features, the ILR is associated with a lower incidence of POPF and with a shortening of postoperative hospital stay with a significant reduction of costs. We believe this is due to the separate drainage of bile and pancreatic juice. In fact, the failure of the bile to activate pancreatic enzymes reduces the tissue damage caused by POPF. When a POPF occurs, it remains nearly pure and the complications are less severe than those caused by the complex POPF where an aggressive mixture is formed from mixed bile and pancreatic juice. ^5^

## Conclusions

In this study, ILR after PD, in patients with soft pancreatic texture and small diameter of the main pancreatic duct, is associated with a lower incidence of POPF and with a shortening of postoperative hospital stay. In conclusion, although ILR cannot be considered as the standard of care, it could be a useful technique in case of a “very soft” pancreas as frequently observed in tumors of the ampulla, distal CBD and duodenum. A prospective randomized study taking into account these variables could provide a reproducible solution of this issue.
